# Supplementary material for: TomoNet: A streamlined cryogenic electron tomography software pipeline with automatic particle picking on flexible lattices
Source: Biol Imaging. 2024 May 9;4:e7. doi: 10.1017/S2633903X24000060 (PMC11140495; doi:10.1017/S2633903X24000060)
Supplement: Wang et al. supplementary material [file S2633903X24000060sup001.zip › Movie legends.docx]

**Movie Legends**

**Movie 1, A spherical VLP consisting of hexamer Gag subunits, colored by local surface curvature. “bad” particles with wrong alignment are shown as red.**

**Movie 2, A VLP lattice with irregular shape.**
